# Pulmonary nodules with explosive calcification were finally diagnosed as lung adenocarcinom: A case report

**DOI:** 10.1097/MD.0000000000046902

**Published:** 2026-01-02

**Authors:** Zhen Li, Min Song, Mingzhen Zhu, Yu Zhang, Luyuan Tian, Hongchuan Zhao

**Affiliations:** a Affiliated Hospital of Jining Medical University, Jining, Shandong Province, China.

**Keywords:** lung adenocarcinoma, medical imaging, case report, pulmonary calcified nodule

## Abstract

**Rationale:**

Pulmonary nodules are a common lung condition, and accurately determining their nature is crucial for doctors to develop appropriate treatment plans. Early identification of malignant nodules can improve patient survival rates. Therefore, accurately distinguishing between benign and malignant pulmonary nodules is essential for optimizing patient management and enhancing the efficiency of healthcare resource utilization.

**Patient concerns:**

The patient is a 53-year-old male. During a routine health examination, a calcified pulmonary nodule was identified. Initially, the nodule was considered benign due to its characteristic calcification and stable appearance on imaging. Over an 8-month follow-up period, serial imaging studies demonstrated no significant changes in the size or morphology of the nodule. Despite the absence of progression, the patient expressed concern regarding the possibility of lung cancer, prompting further diagnostic evaluation.

**Diagnoses:**

Contrast-enhanced computed tomography scan revealed a mass-like high-density shadow in the anterior segment of the left upper lobe, with clear margins, measuring approximately 16 mm × 14 mm × 13 mm. The lesion is adjacent to the trachea and exhibits lobulation, spiculation, and pleural retraction. Calcification is present within the lesion, and blood vessels are seen entering it. The enhanced scan showed mild enhancement. Multiple nodules were observed near the pleura of the left lower lobe. All lung cancer-related tumor markers were negative. The patient underwent a thoracoscopic partial lobectomy (left upper and lower lobes) and thoracoscopic lymph node dissection. Postoperative pathology confirmed invasive lung adenocarcinoma with intrapulmonary metastasis.

**Interventions:**

The patient underwent surgical treatment, followed by genetic testing, which revealed anaplastic lymphoma kinase and AKT serine/threonine kinase 1 mutations. The patient is now receiving targeted therapy with ensartinib.

**Outcomes:**

The patient was discharged 7 days post-surgery and is now on ensartinib for targeted therapy, with regular follow-up examinations scheduled.

**Lessons:**

This case emphasizes that nodules with diffuse, popcorn-like calcification do not rule out the possibility of lung cancer. A comprehensive evaluation integrating multidimensional imaging features is necessary. For nodules with conflicting imaging characteristics, minimally invasive surgical resection remains valuable for diagnosis. This approach broadens our diagnostic perspective on calcified nodules, enhances clinicians’ understanding of lung adenocarcinoma, and reduces the rate of misdiagnosis.

## 1. Introduction

Lung cancer is the leading cause of cancer-related deaths worldwide, accounting for 11.6% of all cancer diagnoses and 18.4% of cancer-related mortality in both men and women globally.^[1]^ Even in the context of early-stage disease, the survival rate of lung cancer patients lags behind that of patients with other common cancers, such as colon, breast, and prostate cancer, with a persistently low 5-year survival rate.^[2]^ Despite advances in diagnostic techniques (e.g., low-dose computed tomography screening) and therapeutic strategies (e.g., targeted therapies and immune checkpoint inhibitors), the incidence and mortality rates of lung cancer remain persistently elevated.^[3]^ Furthermore, treatment-related toxicities – such as immune-related pneumonitis and renal impairment – significantly impair patients’ quality of life.^[4]^ Due to the frequent absence of clinical manifestations in early-stage disease, most patients are diagnosed at advanced stages where surgical intervention is no longer feasible. Consequently, early detection and precise prognostic risk stratification are critical for improving clinical outcomes. In contemporary practice, low-dose computed tomography of the thorax is widely employed for lung cancer screening, with established utility in diagnosing thoracic mass lesions.^[5,6]^ However, reliance solely on imaging characteristics proves insufficient for comprehensive prognostic assessment. Notably, inflammatory status and nutritional parameters play pivotal roles in lung carcinogenesis and progression. There is a growing interest to identify reliable biomarkers to enhance risk stratification and facilitate more personalized treatment escalation in patients with a higher risk of recurrence and treatment de-escalation for patients with a favorable prognosis to reduce toxicity. Although multiple prognostic biomarkers – including programmed death ligand 1 (PD-L1),^[7]^ skeletal muscle index, serum albumin,^[8]^ neutrophil-to-lymphocyte ratio, and neutrophil-to-eosinophil ratio^[9]^ – have been integrated into clinical practice, their applications face inherent limitations. The recently proposed comprehensive prognostic index,^[10]^ which integrates laboratory parameters (e.g., lymphocyte counts, albumin levels) and skeletal muscle index, demonstrates enhanced prognostic utility in patient stratification.

## 2. Case description

Eight months ago, the patient underwent a chest computed tomography (CT) scan during a routine physical examination, which revealed a pulmonary nodule. At that time, the patient had no symptoms such as cough, sputum production, fever, night sweats, chest tightness, or palpitations. The local hospital recommended regular follow-up. Three months later, the patient visited our hospital, and a chest CT scan showed a high-density nodule with calcification in the anterior segment of the left upper lobe. After reviewing the images, the outpatient physician considered it likely to be a benign tumor but could not completely rule out other possibilities. After thorough communication with the patient, regular follow-up was recommended. Three months later, the patient returned for a follow-up, and the chest CT scan showed that the high-density nodule with calcification in the left lung was similar to the previous findings. The patient was advised to continue observation at home. Two months later, the patient returned for another visit, and the chest CT scan still showed no significant changes in the nodule with calcification in the left upper lobe. After detailed communication, the patient agreed to undergo surgical treatment and was admitted to the thoracic surgery department.

The patient has an 8-year history of diabetes mellitus, treated with oral metformin and acarbose, with good glycemic control. The patient denied a history of coronary heart disease, hypertension, or other chronic diseases. The patient has a 30-year smoking history, approximately 10 cigarettes/d, and a history of occasional alcohol consumption. There is no relevant family history. Physical examination: no significant abnormalities were found on systemic examination.

**Figure 1.**
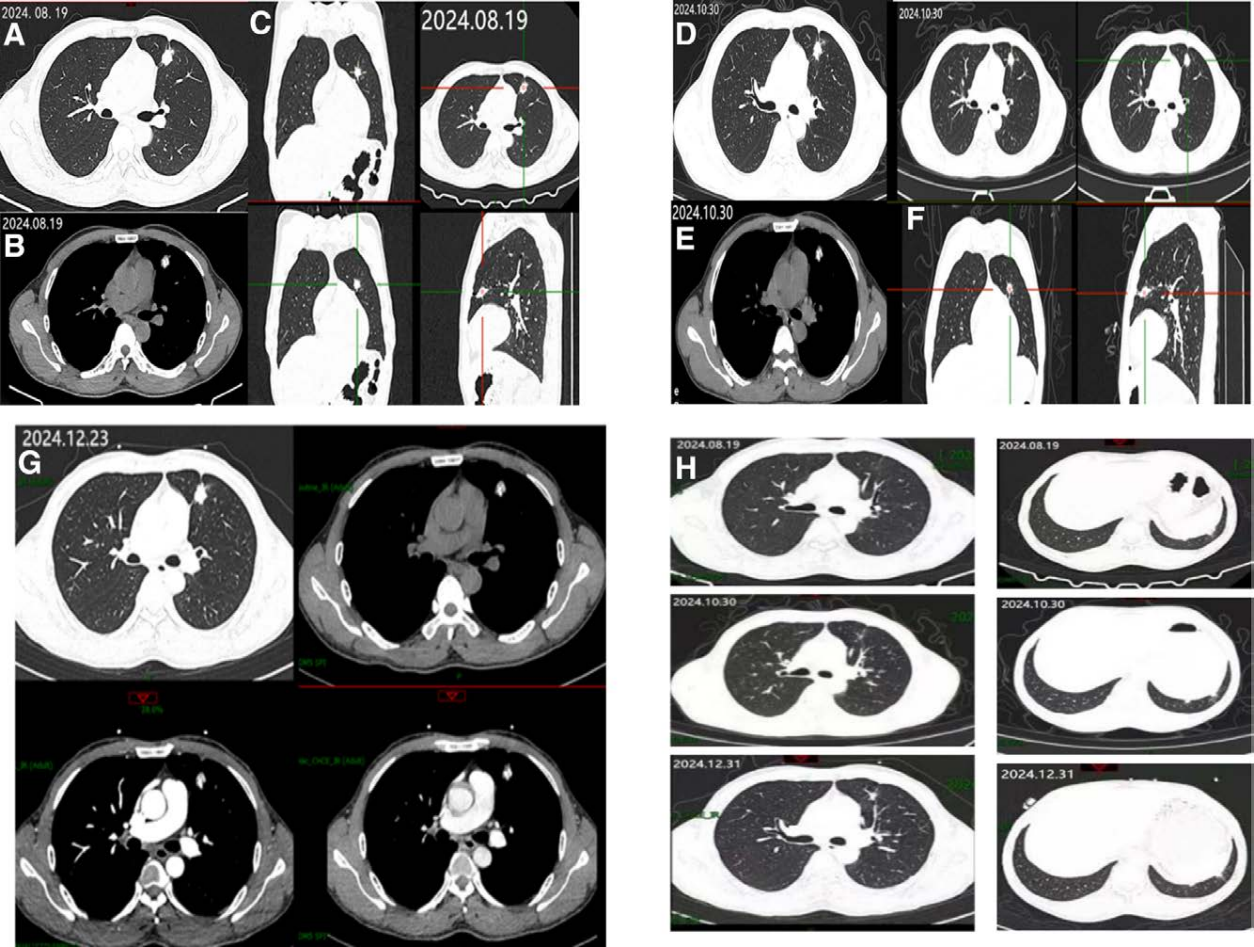
August 19, 2024 CT, (A) lung window, (B) mediastinal window, (C) lung window MPR. October 30, 2024 CT, (D) lung window, (E) mediastinal window, (F) lung window MPR. December 23, 2024 CT, (G) changes in pulmonary window, mediastinal window, arterial phase, delayed phase, (H) changes in oblique fissure nodules in patients. CT = computed tomography, MPR = multi-planar reformation.

**Figure 2.**
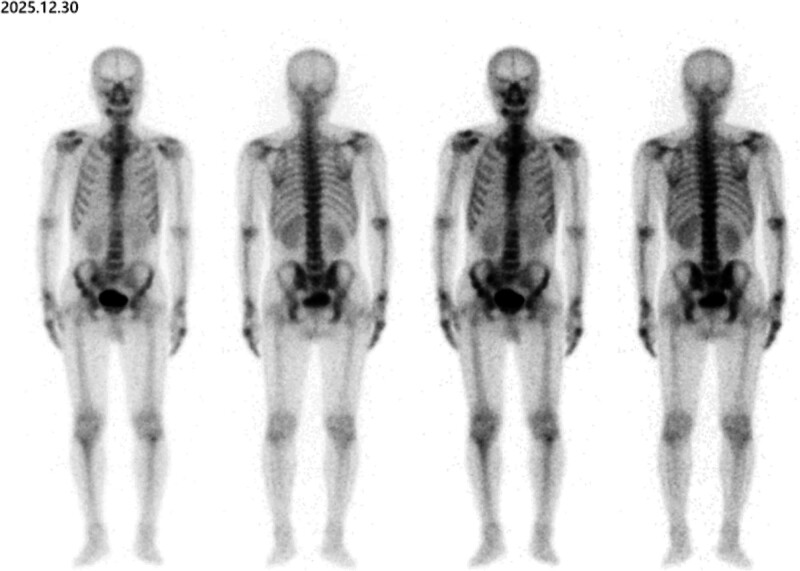
Whole body nuclear imaging of patient on May 30, 2025.

After admission, relevant tests were completed. Laboratory test results were as follows – tumor suppressor gene p53 autoantibody: 0.10 U/mL, protein gene product 9.5 autoantibody: 0.20 U/mL, transcription factor 2 autoantibody: 1.00 U/mL, tumor-associated protein 7 autoantibody: 2.60 U/mL, helicase 4 to 5 autoantibody: 5.50 U/mL, melanoma antigen A1 autoantibody: 0.30 U/mL, tumor-associated gene protein autoantibody: 0.10 U/mL, imaging findings – chest CT scan showed: a high-density shadow with calcification in the anterior segment of the left upper lobe (nodule size approximately 16 mm × 14 mm × 13 mm); a high-density shadow near the pleura of the left lower lobe and nodular thickening of the left interlobar fissure, suggesting possible inflammatory lesions (Fig. 1). Whole body bone imaging shows no signs of bone metastasis (Fig. 2). After excluding surgical contraindications, the patient underwent thoracoscopic partial lobectomy (left upper and lower lobes) + thoracoscopic lymph node dissection. Postoperative management included closed thoracic drainage, fluid replacement, and wound care.

**Figure 3.**
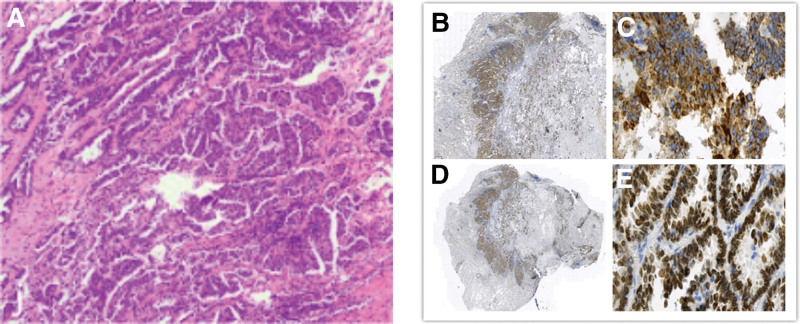
Pathological H&E staining images, (A) gross (B) and microscopic (C) images of pathological napsin A IHC staining, gross (D) and microscopic (E) images of pathological TTF-1 immunohistochemical staining. H&E = hematoxylin and eosin, IHC = immunohistochemical.

Postoperative pathological results revealed: peripheral invasive adenocarcinoma of the left upper lobe (acinar + papillary + micropapillary types), tumor size 18 mm × 10 mm × 11 mm, with alveolar spread and invasion of the visceral pleura; peripheral invasive adenocarcinoma of the left lower lobe (acinar + papillary types), tumor size 15 mm × 10 mm × 3 mm, with alveolar spread and invasion of the visceral pleura; chest wall nodule biopsy showed cancer in fibrous connective tissue and muscle tissue. Immunohistochemistry results: tumor cells were positive for TG, TTF-1, and NapsinA, negative for p40 and Pax-8, and Ki-67 positivity was 20% to 30%. Special staining: (A1, D1) elastic fiber staining (fragmentation +; Fig. 3).

**Figure 4.**
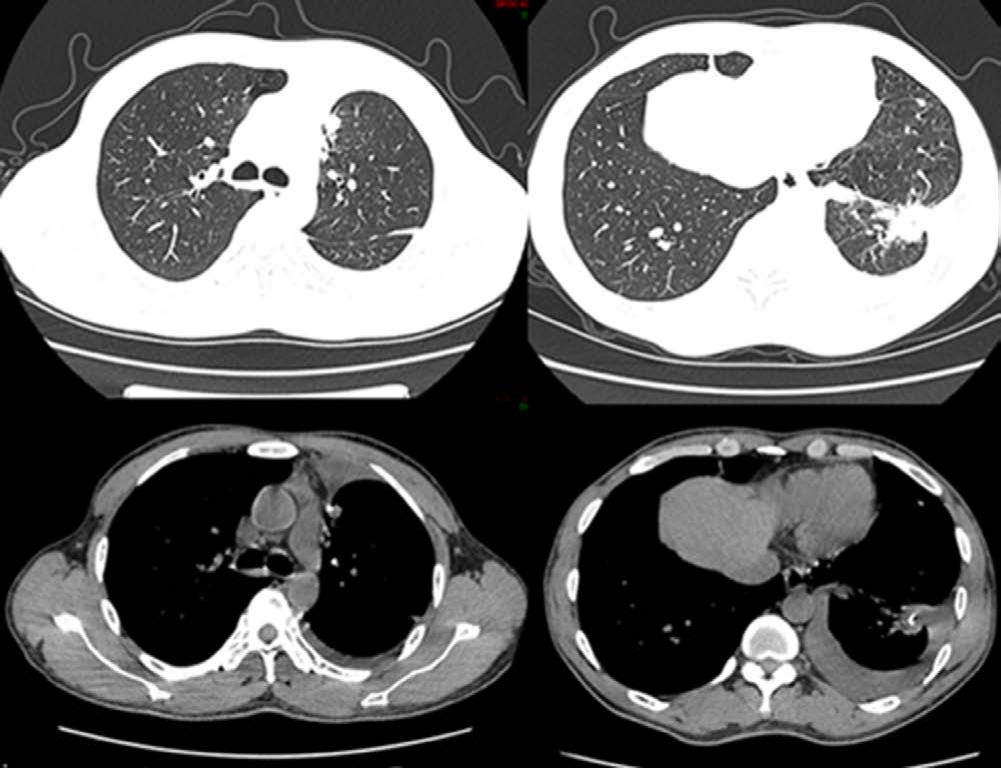
Postoperative follow-up CT scan (January 23, 2025). CT = computed tomography.

On the fourth postoperative day, the closed thoracic drainage showed no gas or fluid output, and a follow-up CT scan indicated good lung re-expansion. The drainage tube was removed. Postoperative genetic testing revealed anaplastic lymphoma kinase and AKT serine/threonine kinase 1 mutations. The patient recovered smoothly and was discharged on the seventh postoperative day. Based on the genetic testing results, the patient was started on ensartinib targeted therapy and scheduled for regular chest CT follow-ups (Fig. 4). The recent follow-up (March 15, 2025) demonstrated favorable recovery, and subsequent follow-up and treatment plans will be managed as lung adenocarcinoma.

## 3. Discussion

Pulmonary adenocarcinoma often originates from the epithelium of smaller bronchi and typically presents as peripheral lung cancer located below the lung segment. Clinically, patients may be asymptomatic or exhibit nonspecific chest symptoms such as dry cough or chest pain. On chest CT, preinvasive lesions often appear round or oval. As the degree of invasion deepens, the lesion pulls on surrounding tissues and is obstructed by blood vessels and bronchi during growth, gradually exhibiting diverse imaging features. Common CT imaging manifestations include solitary nodules or masses, characterized by shape (round, oval, polygonal, lobulated, spiculated, or with spiculation), size, growth rate, density (ground-glass opacity, air bronchogram, vacuole sign, cavitation, calcification [uniform, laminated, punctate]), peripheral features (pleural indentation, bronchial cutoff, vascular convergence), and post-contrast angiographic signs, which are often strongly suggestive of or against lung cancer.^[11]^ In this case, laboratory tests for potential lung cancer biomarkers were all negative, and imaging showed both malignant features (e.g., lobulation, spiculation, pleural indentation) and benign features (e.g., popcorn-like calcification, slow nodule doubling time), making a definitive preoperative diagnosis challenging.

The presence of calcification has long been recognized as one of the most important imaging features for distinguishing benign from malignant pulmonary nodules. A large central core, internal ring, or calcified shell, or complete calcification, is a reliable sign of benign lesions.^[12]^ Calcification is often symmetrically distributed and commonly seen in benign lesions such as tuberculomas or hamartomas. However, malignancy cannot be completely ruled out, although calcification in lung cancer is rare. Studies suggest that typical calcification in lung cancer is fine, granular, amorphous, diffusely distributed, and has low CT values. Smaller calcifications are more suggestive of malignancy. Eccentric calcification with a small number of calcified foci may indicate malignancy, especially needle-like calcification, which is considered an important feature of pulmonary adenocarcinoma. Diffuse, concentric, or popcorn-like calcifications are even rarer.^[13]^

This case first requires differentiation from tuberculoma. Early diagnosis and resection of small lung cancer can significantly improve prognosis, whereas if the nodule is more likely to be a tuberculoma, unnecessary surgery can be avoided.^[14]^ Typical tuberculomas usually appear as round, well-defined lesions with small satellite nodules nearby and coarse calcifications internally.^[15]^ However, differentiating atypical nodules is more challenging, as tuberculomas can also exhibit malignant CT features such as spiculation, pleural indentation, and pleural traction. Studies suggest that tuberculomas tend to have smaller lobulations, fewer pleural indentations, and more satellite nodules and tree-in-bud appearances around the lesion.^[16,17]^ In this case, the lesion exhibited calcification, lobulation, spiculation, and pleural indentation, with diffuse, popcorn-like calcification and a slow doubling rate, showing no significant morphological changes over 8 months of follow-up. These features are more suggestive of tuberculosis. However, calcification in pulmonary adenocarcinoma is typically punctate or fine granular, whereas this case also showed bronchial cutoff and vascular convergence, which are more indicative of malignancy. Additionally, the presence of an air bronchogram is an important indicator of malignancy, particularly adenocarcinoma,^[18]^ but this feature was absent in this case, limiting its diagnostic utility. Furthermore, differentiation from pulmonary hamartoma is necessary. Similar to tuberculoma, early resection of small lung cancer can improve prognosis, whereas hamartomas can avoid unnecessary surgery.^[19]^ The 2 typical features of hamartomas are fat and typical calcification. The popcorn-like or diffuse coarse calcification in this case is more suggestive of hamartoma. According to the “Expert Consensus on Standardized Diagnosis and Treatment of Pulmonary Nodules (2024 Edition),” calcification is classified as a benign feature.^[11]^ However, hamartomas typically appear on chest CT as round or oval nodules with smooth margins and a complete capsule, or as mildly lobulated nodules with uniform or nonuniform density.^[20]^ In this case, the presence of lobulation, spiculation, pleural indentation, bronchial cutoff, and vascular convergence provided reliable evidence for the diagnosis of pulmonary adenocarcinoma.

Another unusual aspect of this case is the nodule’s doubling time. Among lung cancer subtypes, the average volume doubling times for adenocarcinoma, squamous cell carcinoma, and small cell lung cancer are 223 days, 140 days, and 73 days, respectively.^[21]^ However, in this case, the nodule showed no significant growth during follow-up. Studies indicate that a higher proportion of indolent lung cancers are adenocarcinomas, accounting for up to 34.9%,^[21]^ further complicating the diagnosis.

Substantial knowledge gaps persist regarding calcified malignant nodules. First, the pathobiological mechanisms linking calcification – particularly the diffuse “popcorn” pattern – to pulmonary adenocarcinoma remain elusive. Although multiple hypotheses exist, the precise calcification pathways lack definitive experimental validation. Regarding the formation of calcification in nodules, studies suggest that calcification in malignant lesions may result from 3 processes: tumor engulfment of calcified scar tissue or cartilage, dystrophic calcification in necrotic tumors, and primary tumor calcification.^[22]^ This patient had dry pleural metastases, and during follow-up, the nodules along the oblique fissure showed an increasing trend. Studies indicate that the metastasis rate of lung cancer adjacent to the pleura is as high as 37%. Pleural metastases in lung cancer are divided into wet pleural metastases (with malignant pleural effusion) and dry pleural metastases (without pleural effusion). Dry pleural metastases are more common in lung cancers where the primary lesion is adjacent to the pleura or interlobar fissure or exhibits pleural indentation, with approximately 90% of cases being adenocarcinomas. Dry pleural metastases are often ipsilateral, with imaging features including pleural nodules and pleural thickening, the former being more common. Interlobar pleural nodules are particularly noticeable due to the contrast provided by aerated lung tissue. These nodules are typically visible only on lung windows and often appear as bead-like arrangements along the interlobar pleura (usually >6) or clustered around the fissure.^[23]^ Therefore, when CT imaging of non-small cell lung cancer patients reveals pleural nodules (especially multiple interlobar pleural nodules) or pleural thickening, the possibility of dry pleural metastases should be considered to avoid missed diagnoses.

In summary, this case establishes a critical clinical reference point for diagnosing and managing calcified pulmonary nodules. It rectifies the oversimplified traditional view that equates intrapulmonary calcification with benignity, necessitating a redefinition of calcification as a potential malignant indicator. Crucially, it underscores that the presence of diffuse popcorn-like calcification – classically associated with hamartomas – does not preclude lung malignancy. This mandates comprehensive assessment through integration of radiologically multidimensional imaging characteristics to avoid diagnostic delay, thereby ultimately improving patient prognosis. For nodules with conflicting imaging characteristics where malignancy cannot be definitively ruled out, surgical resection remains imperative. Surgical intervention should be considered under the following circumstances: solitary lesions > 2.5 cm in diameter; absence of definitive imaging characteristics excluding malignancy; lesion recurrence or enlargement during follow-up; persistent pulmonary lesions after pharmacotherapy; and cases imposing significant psychological burden on patients. In recent years, minimally invasive thoracic surgery with small incisions has gained increasing preference due to its advantages including reduced intraoperative bleeding, shorter operative duration, minimal impact on pulmonary function, lower complication rates, accelerated recovery, and shorter postoperative hospitalization. In the next 5 years, AI tools, trained on enriched datasets of calcified malignancies, will evolve to quantify composite risk in discordant nodules, reducing diagnostic errors. Liquid biopsy (e.g., ctDNA analysis) may stratify risk in such nodules – detecting oncogenic drivers (e.g., anaplastic lymphoma kinase) would mandate aggressive diagnostic intervention, while negative results could support surveillance. Meanwhile, understanding calcification mechanisms may reveal novel therapeutic targets (e.g., modulating calcium metabolism or associated inflammatory microenvironments) to alter progression in high-risk nodules.

The limitations of this study include: a relatively short follow-up period for the patients; the lack of assessment of patient prognosis; and the absence of an in-depth exploration of the specific mechanisms underlying calcification formation.

## Author contributions

**Conceptualization:** Min Song.

**Data curation:** Hongchuan Zhao.

**Formal analysis:** Mingzhen Zhu.

**Investigation:** Luyuan Tian.

**Project administration:** Yu Zhang.

**Software:** Mingzhen Zhu.

**Validation:** Yu Zhang.

**Visualization:** Min Song, Luyuan Tian.

**Writing – original draft:** Zhen Li.

**Writing – review & editing:** Hongchuan Zhao.

## References

[1] Sung H Ferlay J Siegel RL. Global cancer statistics 2020: GLOBOCAN estimates of incidence and mortality worldwide for 36 cancers in 185 countries. CA Cancer J Clin. 2021;71:209–49. 33538338 10.3322/caac.21660

[2] Forde PM Spicer J Lu S; CheckMate 816 Investigators. Neoadjuvant nivolumab plus chemotherapy in resectable lung cancer. N Engl J Med. 2022;386:1973–85. 35403841 10.1056/NEJMoa2202170 PMC9844511

[3] Bade BC Dela Cruz CS. Lung cancer 2020: epidemiology, etiology, and prevention. Clin Chest Med. 2020;41:1–24. 32008623 10.1016/j.ccm.2019.10.001

[4] Guven DC Erul E Kaygusuz Y. Immune checkpoint inhibitor-related hearing loss: a systematic review and analysis of individual patient data. Support Care Cancer. 2023;31:624. 37819422 10.1007/s00520-023-08083-w

[5] Lee E Kazerooni EA. Lung cancer screening. Semin Respir Crit Care Med. 2022;43:839–50. 36442474 10.1055/s-0042-1757885

[6] Eisenhuber E Schaefer-Prokop C Mostbeck G. [Mimics of lung cancer]. Radiologe. 2019;59:57–70. 30552483 10.1007/s00117-018-0476-3

[7] Rizzo A Dall’Olio FG Altimari A Giunchi F Ardizzoni A. Role of PD-L1 assessment in advanced NSCLC: does it still matter? Anticancer Drugs. 2021;32:1084–5. 34232942 10.1097/CAD.0000000000001118

[8] Guven DC Sahin TK Erul E. The association between albumin levels and survival in patients treated with immune checkpoint inhibitors: a systematic review and meta-analysis. Front Mol Biosci. 2022;9:1039121. 36533070 10.3389/fmolb.2022.1039121 PMC9756377

[9] Sahin TK Ayasun R Rizzo A Guven DC. Prognostic value of neutrophil-to-eosinophil ratio (NER) in cancer: a systematic review and meta-analysis. Cancers. 2024;16:3689. 39518127 10.3390/cancers16213689 PMC11545344

[10] Bas O Sahin TK Karahan L Rizzo A Guven DC. Prognostic significance of the cachexia index (CXI) in patients with cancer: a systematic review and meta-analysis. Clin Nutr ESPEN. 2025;68:240–7. 40157535 10.1016/j.clnesp.2025.03.023

[11] Chinese Thoracic Society, Chinese Medical Association; Chinese Alliance Against Lung Cancer Expert Group. [Chinese expert consensus on diagnosis and treatment of pulmonary nodules(2024)]. Zhonghua Jie He He Hu Xi Za Zhi. 2024;47:716–29. Chinese. 39069848 10.3760/cma.j.cn112147-20231109-00300

[12] Goldstein MS Rush M Johnson P Sprung CL. A calcified adenocarcinoma of the lung with very high CT numbers. Radiology. 1984;150:785–6. 6695080 10.1148/radiology.150.3.6695080

[13] Gao F Ge X Li M. CT features of lung scar cancer. J Thorac Dis. 2015;7:273–80. 25922703 10.3978/j.issn.2072-1439.2015.02.07 PMC4387425

[14] Feng B Chen X Chen Y. Radiomics nomogram for preoperative differentiation of lung tuberculoma from adenocarcinoma in solitary pulmonary solid nodule. Eur J Radiol. 2020;128:109022. 32371184 10.1016/j.ejrad.2020.109022

[15] Zhang J Han T Ren J Jin C Zhang M Guo Y. Discriminating small-sized (2 cm or less), noncalcified, solitary pulmonary tuberculoma and solid lung adenocarcinoma in tuberculosis-endemic areas. Diagnostics (Basel). 2021;11:930. 34064284 10.3390/diagnostics11060930 PMC8224307

[16] Zhuo Y Zhan Y Zhang Z. Clinical and CT radiomics nomogram for preoperative differentiation of pulmonary adenocarcinoma from tuberculoma in solitary solid nodule. Front Oncol. 2021;11:701598. 34712605 10.3389/fonc.2021.701598 PMC8546326

[17] Pillay T Andronikou S Zar HJ. Chest imaging in paediatric pulmonary TB. Paediatr Respir Rev. 2020;36:65–72. 33160839 10.1016/j.prrv.2020.10.002

[18] Totanarungroj K Chaopotong S Tongdee T. Distinguishing small primary lung cancer from pulmonary tuberculoma using 64-slices multidetector CT. J Med Assoc Thai. 2012;95:574–82. 22612014

[19] Lundeen KS Raj MS Rajasurya V Sharma S Ludhwani D. Pulmonary Hamartoma. In: *StatPearls*. StatPearls Publishing; 2025. https://pm.yuntsg.com/details.html?pmid=30969628. Accessed March 17, 2025 30969628

[20] Li B Xin Z Xue W Zhang X. Lung hamartoma resembling lung cancer: a report of three cases. J Int Med Res. 2022;50:3000605221132979. 36324241 10.1177/03000605221132979 PMC9634196

[21] Jiang B Han D van der Aalst CM. Lung cancer volume doubling time by computed tomography: a systematic review and meta-analysis. Eur J Cancer. 2024;212:114339. 39368222 10.1016/j.ejca.2024.114339

[22] Stewart JG MacMahon H Vyborny CJ Pollak ER. Dystrophic calcification in carcinoma of the lung: demonstration by CT. AJR Am J Roentgenol. 1987;148:29–30. 3491520 10.2214/ajr.148.1.29

[23] Wei SH Zhang JM Shi B Gao F Zhang ZX Qian LT. The value of CT radiomics features to predict visceral pleural invasion in ≤3 cm peripheral type early non-small cell lung cancer. J X-Ray Sci Technol. 2022;30:1115–26. 10.3233/XST-221220 35938237

